# Diagnostic performance of neuroimaging modalities for epileptogenic focus localization: A systematic review

**DOI:** 10.1002/epi4.70178

**Published:** 2025-11-10

**Authors:** Mustafa S. Alhasan, Mohammed Khalil, Ayman S. Alhasan, Ahmed Najjar, Yasir Hassan Elhassan, Abdullah Almaghraby, Omar Alharthi, Seham Hamoud, Muhammed Amir Essibayi, Fabricio Feltrin, Sumit Singh, James Milburn, Ahmed Y. Azzam

**Affiliations:** ^1^ Department of Internal Medicine College of Medicine, Taibah University Madinah Saudi Arabia; ^2^ Teleradiology Solutions Ardmore Pennsylvania USA; ^3^ Faculty of Medicine King Abdulaziz University Jeddah Saudi Arabia; ^4^ Department of General and Specialized Surgery Taibah University Madinah Saudi Arabia; ^5^ Department of Basic Medical Sciences College of Medicine, Taibah University Madinah Saudi Arabia; ^6^ Department of Pediatrics Umm Al‐Qura University Makkah Saudi Arabia; ^7^ Department of Pediatrics Taibah University Madinah Saudi Arabia; ^8^ Montefiore‐Einstein Cerebrovascular Research Lab Montefiore Medical Center, Albert Einstein College of Medicine Bronx New York USA; ^9^ Department of Neurological Surgery Montefiore Medical Center, Albert Einstein College of Medicine Bronx New York USA; ^10^ Division of Radiology—Neuroradiology University of Texas Southwestern Medical Center Dallas Texas USA; ^11^ The University of Queensland Medical School, Ochsner Clinical School New Orleans Louisiana USA; ^12^ Department of Radiology Ochsner Clinic Foundation New Orleans Louisiana USA; ^13^ Clinical Research and Clinical Artificial Intelligence ASIDE Healthcare Lewes Delaware USA; ^14^ Division of Global Health and Public Health, School of Nursing, Midwifery and Public Health University of Suffolk Ipswich UK

**Keywords:** epilepsy, multimodal imaging, neuroimaging, radiosurgery, stereotactic

## Abstract

**Objective:**

Accurate localization of epileptogenic foci remains of significant importance for surgical planning in drug‐resistant epilepsy. Multiple neuroimaging modalities are available; however, their comparative diagnostic performance lacks comparative detailed synthesis. This systematic review aimed to evaluate and compare the diagnostic accuracy of structural MRI, PET imaging, SPECT/SISCOM, and combined multimodal strategies for epileptogenic focus localization.

**Methods:**

We conducted a systematic review following PRISMA 2020 guidelines, searching PubMed, Scopus, Google Scholar, Cochrane Library, and Web of Science databases up to May 30, 2025. Studies evaluating the diagnostic performance of neuroimaging modalities for epilepsy focus localization with surgical correlation were included. Data extraction focused on sensitivity, specificity, and clinical manner. Quality assessment used QUADAS‐2 criteria.

**Results:**

Fifteen studies included a total of 1157 patients that met inclusion criteria. Combined multimodal strategies integrating two or more imaging modalities demonstrated the highest diagnostic performance (sensitivity 82–100%), followed by structural MRI in lesional epilepsy (72–100% sensitivity). PET imaging showed consistent performance across clinical contexts (33–89% sensitivity), while SPECT/SISCOM exhibited variable results (33–83% sensitivity). Strong complementarity existed between MRI and PET (85% concordance), with context‐dependent optimization for lesional versus non‐lesional epilepsy.

**Significance:**

Combined multimodal neuroimaging provides superior diagnostic performance for epileptogenic focus localization. Clinical context significantly impacts the modality selection, with MRI prioritized in lesional cases and functional imaging essential for MRI‐negative epilepsy. These findings support evidence‐based imaging protocols for surgical epilepsy evaluation.

**Plain Language Summary:**

This systematic review evaluated which brain imaging techniques are best for finding the exact location where seizures start in people with drug‐resistant epilepsy who need surgery. The researchers analyzed 15 studies involving 1157 patients. They found that using multiple imaging techniques together (combining structural and functional imaging) provides the most accurate results, with success rates of 82–100%. Standard MRI scans work very well (72–100% accuracy) when there is a visible brain abnormality causing seizures. However, for patients whose MRI looks normal, additional functional imaging techniques like PET or SPECT scans are crucial, achieving 63–89% accuracy. The study shows that the best imaging approach depends on the individual patient's situation: MRI should be used first when a brain lesion is suspected, but functional imaging becomes essential when MRI does not show anything abnormal. These findings help doctors choose the right combination of imaging tests for each patient to improve surgical planning and outcomes.

**Key points**

- Combined multimodal imaging (82–100% sensitivity) outperforms single modalities for localizing epileptogenic foci in surgical candidates.
- MRI excels in lesional epilepsy (72–100% sensitivity) but functional imaging is essential for MRI‐negative cases (63–89% sensitivity).
- Study reviewed 15 studies with 1157 patients undergoing presurgical epilepsy evaluation with neuroimaging and surgical correlation.
- PET and SPECT/SISCOM show moderate but variable performance (33–89% and 33–83% sensitivity, respectively) across clinical contexts.
- Imaging strategy should be context‐dependent: MRI‐first for lesional cases, add functional imaging for non‐lesional epilepsy.

## 1 INTRODUCTION

Epilepsy affects around 65 million individuals around the world, with almost 30% of patients developing drug‐resistant epilepsy requiring consideration for surgical intervention. Successful epilepsy surgery depends significantly on accurate localization of the epileptogenic zone, defined as the brain region where seizures originate and whose removal or disconnection is necessary for seizure freedom. This localization process represents one of the most challenging aspects of presurgical evaluation, requiring integration of multiple diagnostic modalities to achieve the best surgical outcomes possible.^1^, ^2^, ^3^

The recent literature evidence about epilepsy surgery evaluation relies heavily on advanced neuroimaging techniques to identify and characterize epileptogenic foci. Structural magnetic resonance imaging (MRI) serves as the cornerstone of epilepsy imaging, providing detailed anatomical information about the possible epileptogenic lesions including hippocampal sclerosis, focal cortical dysplasia, and other structural abnormalities. However, around 20–30% of surgical candidates present with MRI‐negative epilepsy, necessitating functional neuroimaging approaches to identify subtle metabolic or perfusion abnormalities associated with epileptogenic tissue.^4^, ^5^

Positron emission tomography (PET) imaging, especially with fluorodeoxyglucose (FDG), has emerged as a powerful tool for detecting interictal hypometabolism in epileptogenic regions, demonstrating special value in temporal lobe epilepsy and MRI‐negative cases. Single‐photon emission computed tomography (SPECT), especially when performed with subtraction ictal‐interictal SPECT co‐registered to MRI (SISCOM), provides advanced information about seizure‐related perfusion changes, offering valuable highlights and information into ictal propagation patterns and seizure onset zones.^6^, ^7^

The advancement toward multimodal imaging strategies reflects recognition that individual modalities possess limitations and that combined strategies may provide superior diagnostic accuracy. However, the best selection and sequencing of neuroimaging modalities remain poorly defined, with significant variability in practice settings across epilepsy centers. This variability originates partly from limited high‐quality comparative evidence synthesizing the diagnostic performance of different imaging strategies across different clinical manners.^8^, ^9^, ^10^, ^11^

Previous studies have looked at the individual neuroimaging modalities in isolation, but a comparative detailed overview targeted at the relative performance and advancements of different imaging approaches remains significantly limited. Furthermore, the clinical manner of lesional versus non‐lesional epilepsy significantly influences diagnostic strategies; however, this important distinction has not been addressed.^4^, ^11^, ^12^, ^13^, ^14^, ^15^, ^16^

The present systematic review aims to address these gaps by investigating, evaluating, and comparing the diagnostic accuracy of structural MRI, PET imaging, SPECT/SISCOM, and combined multimodal approaches for epileptogenic focus localization. Our objectives were to determine the diagnostic performance characteristics of each imaging modality, investigate the concordance and complementarity between different approaches, identify the best imaging strategies based on clinical context, and provide an evidence‐based summary for neuroimaging protocols in presurgical epilepsy evaluation.

While electrophysiological methods including video‐electroencephalography (VEEG) monitoring and invasive intracranial recordings remain cornerstone components of detailed presurgical evaluation, this systematic review focuses on the diagnostic performance of neuroimaging modalities, including structural MRI, PET, and SPECT/SISCOM and their multimodal combinations. Electrophysiological data were considered only when serving as reference standards or as part of integrated evaluation protocols in included studies.

## 2 METHODS

### 2.1 Search strategy and information sources

This systematic review was conducted according to the Preferred Reporting Items for Systematic Reviews and Meta‐Analyses (PRISMA) 2020 guidelines.^17^ We performed a literature search of multiple electronic databases including PubMed, Scopus, Google Scholar, Cochrane Library, and Web of Science from database inception up to May 30, 2025.

The literature search strategy has included the following key terms and their variations: (“epilepsy” OR “seizure” OR “epileptogenic” OR “ictal” OR “interictal”) AND (“neuroimaging” OR “brain imaging” OR “magnetic resonance imaging” OR “MRI” OR “positron emission tomography” OR “PET” OR “single photon emission computed tomography” OR “SPECT” OR “SISCOM” OR “functional imaging” OR “multimodal imaging”) AND (“focus localization” OR “seizure focus” OR “epileptogenic zone” OR “surgical planning” OR “presurgical evaluation”) AND (“diagnostic accuracy” OR “sensitivity” OR “specificity” OR “performance” OR “concordance” OR “correlation”) AND (“surgery” OR “surgical outcome” OR “histopathology” OR “pathological confirmation” OR “gold standard”). Additional searches were performed using reference lists of included studies and relevant review articles to identify any missed publications that may be included.

### 2.2 Imaging modality definitions

For standardization across included studies, we categorized imaging modalities as follows: structural MRI was defined as sequences providing anatomical information about brain structure, including T1‐weighted imaging (T1WI: sagittal, axial, coronal; both 2D and 3D acquisitions including MPRAGE and TFE sequences), T2‐weighted imaging (T2WI: fast spin‐echo [FSE] and turbo spin‐echo [TSE] variants), Fluid‐Attenuated Inversion Recovery (FLAIR: axial and coronal), Inversion Recovery (IR) sequences, and volumetric or high‐resolution acquisitions for hippocampal or cortical analysis. Functional neuroimaging modalities included techniques assessing brain metabolism or perfusion, FDG‐PET, SPECT, SISCOM, and resting‐state functional MRI (RS‐fMRI) measuring blood oxygen level‐dependent signal changes. Electrophysiological methods (VEEG and invasive intracranial EEG) were included in data extraction only when studies utilized them as reference standards or as part of integrated multimodal evaluation protocols. While electrophysiological recordings remain essential for presurgical evaluation, this systematic review focused specifically on the diagnostic performance of neuroimaging modalities. Electrophysiological data were considered only in their supporting role for defining reference standards or validating imaging findings.

### 2.3 Eligibility criteria and study selection

Studies were included if they evaluated the diagnostic performance of neuroimaging modalities for epileptogenic focus localization in patients undergoing epilepsy surgery, provided surgical correlation or histopathological confirmation as the reference standard, reported sufficient data to calculate diagnostic accuracy measures including sensitivity and specificity, and were published in peer‐reviewed journals in the English language. We included studies about structural MRI, PET imaging, SPECT/SISCOM, or combined multimodal imaging in adult and pediatric populations with drug‐resistant epilepsy.

Exclusion criteria were defined as case reports and case series with fewer than 10 patients, studies lacking surgical correlation or adequate reference standard, articles focusing only on technical aspects without clinical correlation, duplicate publications or overlapping patient populations, and studies with insufficient data for extraction of diagnostic performance measures. Conference abstracts, editorials, and review articles were excluded, despite our looking at their reference lists to screen for relevant studies that may be included in our study.

We first conducted the initial screening of titles and abstracts, followed by a full‐text review of preliminarily eligible studies. The study selection process was documented using a data extraction sheet.

Lesional epilepsy was operationally defined as cases where any imaging modality (including but not limited to MRI) identified a structural abnormality that was subsequently confirmed by the reference standard (histopathology, surgical correlation, or comprehensive evaluation). Non‐lesional epilepsy specifically referred to cases where conventional structural imaging, mainly MRI, failed to identify visible structural abnormalities; however, the epileptogenic zone was successfully localized through alternative methods including functional imaging, invasive intracranial EEG, or finally confirmed by a favorable surgical outcome. Importantly, the terms “MRI‐negative” and “non‐lesional” are related but not synonymous; MRI‐negative specifically indicates no visible abnormality on MRI interpretation, while non‐lesional epilepsy is finally defined by the absence of a confirmed structural lesion per the reference standard. This distinction is clinically relevant because some MRI‐negative cases may harbor subtle structural abnormalities detectable by other advanced imaging techniques or become apparent only on histopathological examination.

Combined multimodal imaging strategies were defined as diagnostic approaches utilizing two or more imaging modalities in an integrated fashion for epileptogenic focus localization, with prospective synthesis of findings to reach consensus localization. The key distinction between combined and sequential approaches is that combined strategies included prospective integration of findings from multiple modalities to inform decision‐making, rather than simply performing modalities sequentially without structured synthesis. Studies were categorized as utilizing combined approaches only when they specifically reported diagnostic performance metrics based on two or more modalities used together for consensus localization.

### 2.4 Data extraction and quality assessment

Data extraction was performed using a custom data extraction sheet designed for diagnostic accuracy studies. Extracted information included study characteristics (first author, publication year, country, study design, sample size), patient demographics (age, sex, epilepsy type, seizure frequency), imaging parameters (field strength, acquisition protocols, analysis methods), reference standard details (surgical approach, histopathological findings, follow‐up duration), and diagnostic performance measures (sensitivity, specificity, positive and negative predictive values, accuracy). Additional variables included clinical context classification (lesional vs non‐lesional epilepsy), concordance data between imaging modalities, and any reported adverse events or limitations.

Quality assessment was conducted using the Quality Assessment of Diagnostic Accuracy Studies (QUADAS‐2) tool, which evaluates four key domains: patient selection, index test, reference standard, and flow and timing. Each domain was assessed for risk of bias and applicability concerns using predefined signaling questions. Studies were not excluded based on quality assessment results, but quality ratings were incorporated into sensitivity analyses and interpretation of findings.

### 2.5 Network assessment methodology

We conducted a qualitative network analysis to characterize relationships and hierarchical performance observations and patterns among imaging modalities. This included calculating network centrality scores (range between zero and one) representing each modality's relative importance based on three weighted components: (a) frequency of utilization across included studies, (b) weighted average diagnostic performance (sensitivity and specificity), and (c) number and strength of significant concordance relationships with other modalities as reported in included studies. Concordance patterns between modalities were mapped using reported kappa statistics and percentage agreement values. Context‐dependent imaging pathways (lesional vs non‐lesional epilepsy) were identified based on modality progression patterns and performance stratification described in included studies. This approach is analogous to network meta‐analysis visualization frameworks but adapted for diagnostic accuracy synthesis, allowing graphical representation of relative modality performance hierarchies and interdependencies. Network centrality scores were calculated as composite descriptive measures for visualization purposes and do not represent formal statistical network meta‐analysis with indirect comparisons.

## 3 RESULTS

### 3.1 Study selection and characteristics

**FIGURE 1:**
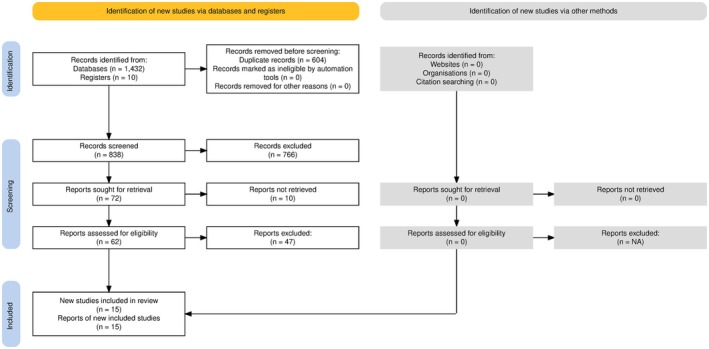
PRISMA flowchart of included studies pipeline.

Following the systematic search strategy, 1442 records were initially identified from electronic databases and registers. After removing 604 duplicates and screening 838 records, 72 reports were sought for retrieval, with 62 reports assessed for full‐text eligibility. We then included a final number of 15 studies that met the inclusion criteria and were included in this systematic review (Figure 1).

### 3.2 Study characteristics and patient demographics

The included studies included a total of 1157 patients across 15 studies conducted between 1995 and 2023 (Table 1). The majority of studies were retrospective in design (12/15, 80%), with two prospective studies and one comparative cohort study. Sample sizes ranged from 18 to 161 patients, with a median of 54 participants. Patient populations demonstrated heterogeneity, with mean ages ranging from 8 to 33.2 years and male representation varying from 38.8% to 73.8%. The studies included diverse epilepsy types, including mixed focal epilepsy, temporal lobe epilepsy, focal cortical dysplasia, and drug‐resistant epilepsy populations. Follow‐up duration varied were reported, ranging from 12 months to a median of 32 months postoperatively.

**TABLE 1:** Study characteristics and patient demographics.

Study	Country	Design	Sample size	Mean age (years)	Male (%)	Epilepsy type	Follow‐up duration
Schulte et al. 2023^18^	Germany	Retro	161	32.3	62.7	Mixed focal epilepsy	Median 32 mo postop
Kikuchi et al. 2021^19^	Japan	Prosp	31	Median 31	54.8	Focal epilepsy (surgical)	NR
Yokota et al. 2020^20^	USA	Retro	62	18.9	45.2	FCD Type I or II	1 year postsurg
Oldan et al. 2018^21^	USA	Retro	74	31	NR	Refractory focal onset epilepsy	NR
Halac et al. 2017^22^	Turkey	Retro	71	21.93 (at surg)	40.8	Drug‐resistant FCD (Type I or II)	NR
Chen et al. 2017^23^	China	Prosp/Retro	42	24 (*n* = 36 EZ)	57.1	Focal epilepsy	3 years (surg cases)
Perissinotti et al. 2014^24^	Spain	Retro	54	8	53.7	Drug‐resistant focal epilepsy (peds)	12 months (*n* = 14); 6 months (*n* = 4)
Gok et al. 2013^25^	USA	Retro	98	~32 (at op)	38.8	Refractory TLE (surgical)	MRI+: 23.4; MRIeq: 21.9; MRI‐: 25.1 months
Kim et al. 2009^26^	Korea	Retro	42	16.42 (at surg)	73.8	Focal epilepsy (pediatric, Engel I)	Mean 2.15 years (0.59–5.05)
Salamon et al. 2008^27^	USA	Comp Cohorts	45	Type I: 16.9; Type II: 8.9	44.4	Cortical dysplasia	Mean 2.0 years
Hong et al. 2002^28^	Korea	Retro	41	28.0 (at surg)	65.9	Non‐lesional neocortical epilepsy	Mean 2.77 years
Won et al. 1999^29^	Korea	Retro	118	27	62.7	Medically intractable epilepsy	Mean 24 months (12–35)
Spanaki et al. 1999^30^	USA	Retro	53	33.2	47.2	Refractory partial epilepsy	Mean 17 months (surg *n* = 24)
Salanova et al. 1998^31^	USA	Retro	38	30 (at surg)	44.7	Medically refractory TLE	Mean 23.9 months (12–40)
Gaillard et al. 1995^32^	USA	Retro	18	29	61.1	CPS temporal lobe origin	Mean 28 months (11–38) (*n* = 9)

Abbreviations: Comp Cohorts, comparative cohorts; CPS, complex partial seizures; EZ, epileptogenic zone; FCD, focal cortical dysplasia; MRI‐, MRI negative; MRI+, MRI positive; MRIeq, MRI equivocal; NR, not reported; peds, pediatric; Prosp, prospective; Retro, retrospective; surg, surgery; TLE, temporal lobe epilepsy.

Reference standards utilized across included studies demonstrated methodological differences reflective of real‐world practice. Histopathological confirmation served as the primary reference standard in eight studies of 15 studies (53%), surgical outcome assessment using Engel classification was utilized in seven studies (47%), and detailed epileptogenic zone determination through multimodal evaluation including invasive EEG monitoring and clinical correlation was utilized in five studies (33%). Some studies utilized multiple reference standards. This heterogeneity in reference standards was evaluated in our quality assessment using QUADAS‐2 criteria later in results.

Regarding combined multimodal imaging approaches, specific combinations utilized across studies included, MRI plus PET (seven studies, most common), MRI plus SPECT/SISCOM (six studies), MRI plus PET plus SPECT (three studies), hybrid PET/MRI simultaneous acquisition (two studies: Kikuchi et al. 2021 and Oldan et al. 2018), and MRI plus functional imaging plus EEG correlation (five studies). These combinations allowed for the assessment of integrated multimodal diagnostic performance as detailed in further results sections.

### 3.3 Imaging protocols and technical parameters

Technical imaging parameters showed significant variability across studies (Table 2). MRI field strengths ranged from 1.0 T to 3.0 T, with most recent studies utilizing 3.0 T systems. Structural MRI protocols have mostly included T1‐weighted, T2‐weighted, and FLAIR sequences, with several studies having specialized epilepsy protocols and high‐resolution volumetric acquisitions. Functional imaging modalities included FDG‐PET (12 studies), ictal and interictal SPECT (eight studies), and SISCOM (six studies). Reader configurations varied from single readers to multiple specialist interpreters, with nuclear medicine physicians, neuroradiologists, and neurosurgeons contributing to image interpretation. Importantly, 12 of 15 studies (80%) had blinded interpretation protocols, improving the reliability of diagnostic accuracy assessments.

**TABLE 2:** Imaging protocols and technical parameters.

Study	MRI field strength	MRI sequences	Functional Imaging Modality and Electrophysiological Methods	Functional imaging protocol	Reader blinding	Number of readers	Same patient cohort	Reference standard
Schulte et al. 2023^18^	NR	NR	Ictal SPECT (VSA and ISAS), EEG, FDG‐PET	Ictal and interictal SPECT; ISAS postprocessing	NR	1 (VSA)	Yes	Clinical focus hypothesis. Surg subgroup: resection site and outcome
Kikuchi et al. 2021^19^	3 T	Conventional + dedicated epilepsy MR protocols (FLAIR)	FDG‐PET/MRI, FDG‐PET/CT, Standalone MRI	PET/CT then PET/MRI. Standalone MRI after	Yes (blinded)	5	Yes	Histopathologically confirmed EZ via resection
Yokota et al. 2020^20^	1.5 T or 3 T	3D T1 cor, 2D T2 ax/cor, 2D FLAIR ax/cor	FDG‐PET	Non‐ictal FDG‐PET	Blinded (visual)	2	Yes	Pathological diagnosis of FCD
Oldan et al. 2018^21^	3 T	Standard epilepsy protocol	FDG‐PET (part of PET/MR)	Hybrid PET/MR, FDG	Yes (blindly re‐int)	2 (MRI), 2 (NM)	Yes	Surgical outcome (Engel I‐III for 24 surg/RNS pts)
Halac et al. 2017^22^	1.5 T (3 T opt)	T1, T2, FLAIR	FDG‐PET	Interictal FDG‐PET	Yes (visual re‐eval)	1 neurosurg, 2 NM	Yes	Histopathological diagnosis of FCD
Chen et al. 2017^23^	3.0 T	Routine + hi‐res cor T1IR, T2TSE, FLAIR (hippocampus)	RS‐fMRI, MRS, VEEG, PET‐CT (FDG)	RS‐fMRI standard. MRS single‐voxel. VEEG std. PET‐CT FDG	Yes (independent)	1 neuro, 1 neurosurg, 1 elec spec, 1 neurorad, 1 NM	Yes	Comprehensive evaluation‐defined EZ
Perissinotti et al. 2014^24^	1.5 T	Specific epilepsy protocol	SISCOM, Interictal FDG‐PET	SISCOM: ictal/interictal SPECT. PET: interictal FDG	Yes (masked)	2	Yes	PEZ by VEEG & clinical data. Surg pts.: path & outcome
Gok et al. 2013^25^	1.5 T or 3 T	3D T1 vol, cor FSE T2, cor FLAIR, cor IR	Interictal FDG‐PET	Interictal FDG‐PET	Yes (MRI, PET, EEG)	1 (MRI), 1 (PET)	Yes	Surgical outcome, Histopathology
Kim et al. 2009^26^	3.0 T	T1 sag, T2 ax, FLAIR ax, oblique cor (T2, FLAIR), 3D T1 TFE cor	FDG‐PET, SISCOM	PET: interictal FDG. SISCOM: ictal/interictal ECD‐SPECT subtracted and coreg. MRI	NR	NR	Yes	Surgical outcome (Engel class I)
Salamon et al. 2008^27^	NR	UCLA MRI epilepsy protocol	FDG‐PET, FDG‐PET/MRI coreg	FDG‐PET. Coregistration	Yes (blindly reviewed)	NR	Partial (comparative cohorts)	Histopathology (CD)
Hong et al. 2002^28^	1.0 T or 1.5 T	T1 sag, T2 ax/cor. Opt: T1 3D MPRAGE, T2 FLAIR	FDG‐PET, Ictal SPECT, Ictal Scalp EEG	PET: interictal FDG. SPECT: ictal 99mTc‐HMPAO	Yes (PET/SPECT)	1 (PET/SPECT)	Yes	Good surgical outcome (Engel 1–3)
Won et al. 1999^29^	1.0 T or 1.5 T	T1 sag, T2 ax/cor; T2 FSE (temporal), T1 3D MPRAGE (temporal)	FDG‐PET, Ictal SPECT	PET: interictal FDG. SPECT: ictal 99mTc‐HMPAO, inj <30s post onset	Yes (pathology)	NR	Yes	Pathologic diagnosis (primary for sens.)
Spanaki et al. 1999^30^	NR	Multiplanar MRI, quantitative measures	Quant. Diff. SPECT, PET, iEEG	SPECT: 99mTc‐HMPAO subtraction. PET: FDG	Yes (diff‐img interp)	3	Yes	iEEG (*n* = 26 for SPECT). Overall surgical localization
Salanova et al. 1998^31^	1.5 T	SE T1 Ax, FSE T2 Ax, FSE T2 Cor	FDG‐PET, Ictal SPECT	Interictal FDG‐PET. Ictal SPECT	Yes (MRI, PET)	1 (MRI), 1–2 (PET)	Yes	Surgical outcome, Pathology
Gaillard et al. 1995^32^	1.5 T	T2‐w MRI, volumetric MRI (hippocampus)	Interictal FDG‐PET	Interictal FDG‐PET	NR	1	Yes	Ictal focus by VEEG

*Note*: EEG modalities listed (VEEG, iEEG, scalp EEG) were incorporated by specific studies as reference standards or for integrated multimodal analysis (Chen et al. 2017, Hong et al. 2002, Spanaki et al. 1999, Perissinotti et al. 201) but were not the primary focus of this systematic review's diagnostic accuracy assessment. This review specifically evaluated neuroimaging modalities (MRI, PET, and SPECT/SISCOM). Electrophysiological data were considered only when serving as reference standards or within multimodal evaluation protocols.

Abbreviations: 2D, two‐dimensional; 3D, three‐dimensional; 99mTc‐HMPAO, technetium‐99 m hexamethylpropyleneamine oxime; ax, axial; CD, cortical dysplasia; cor, coronal; coreg, coregistered; diff‐img interp, difference image interpretation; ECD, ethyl cysteinate dimer; elec spec, electrophysiology specialist; EZ, epileptogenic zone; FCD, focal cortical dysplasia; FDG, fluorodeoxyglucose; FLAIR, fluid‐attenuated inversion recovery; FSE, fast spin echo; hi‐res, high‐resolution; iEEG, intracranial EEG; inj, injection; IR, inversion recovery; ISAS, Ictal‐interictal SPECT analysis by statistical parametric mapping; MPRAGE, magnetization prepared rapid acquisition gradient echo; MRS, magnetic resonance spectroscopy; neuro, neurologist; neurorad, neuroradiologist; neurosurg, neurosurgeon; NM, nuclear medicine; NR, not reported. opt, optional; path, pathology; PET, positron emission tomography; PEZ, presumed epileptogenic zone; re‐int, reinterpreted; RS‐fMRI, resting‐state functional MRI; sag, sagittal; SE, spin echo; sens, sensitivity; SISCOM, subtraction ictal SPECT coregistered to MRI; SPECT, single photon emission computed tomography; surg, surgical; T, Tesla; T1, T1‐weighted imaging; T2, T2‐weighted imaging; TFE, turbo field echo; TSE, turbo spin echo; VEEG, video electroencephalography; vol, volumetric; VSA, visual subtraction analysis.

### 3.4 Reference standards and study framework

The Population, Intervention, Comparator, Outcomes and Setting (PICOS) framework proposed a diverse overview to defining positive imaging findings and reference standards (Table 3). MRI‐positive definitions ranged from visible focal cortical dysplasia to complex categorizations based on epileptogenic zone identification. Functional imaging positivity criteria similarly varied, including visual detection of hypometabolic regions, concordance with clinical focus hypotheses, and anatomical localization based on operative reports. Reference standards demonstrated appropriate rigor, with most studies utilizing histopathological confirmation (eight studies), surgical outcomes assessment (seven studies), or comprehensive evaluation‐defined epileptogenic zones (five studies). This methodological diversity reflects the complexity of epilepsy focus localization but may contribute to between‐study heterogeneity.

**TABLE 3:** PICOS framework and reference standards.

Study	Population (P)	Index test—MRI‐positive definition (I)	Comparator—functional imaging positive definition (C)	Outcomes—reference standard (O)	Study design (S)
Schulte et al. 2023^18^	Presurgical eval (2008–2020), ictal and interictal SPECT and ISAS	MR‐positive: potentially epileptogenic lesion diagnosed in MRI	VSA/ISAS: Concordance with clinical focus hypothesis (Cat A, B, C, D, E)	Clinical focus hypothesis. Surg subgroup: resection site and outcome	Retro
Kikuchi et al. 2021^19^	Focal epilepsy, surgical resection for EZ, FDG‐PET/CT then FDG‐PET/MRI	Detection of EZ (laterality and anatomical part) based on operative report as ref.	Detection of EZ (laterality and anatomical part) based on operative report as ref.	Histopathologically confirmed EZ via resection	Prosp
Yokota et al. 2020^20^	Surgically treated, pathologically diagnosed FCD type I or II	Visual delineation of abnormal regions on MRI (implying FCD)	Visual delineation of hypometabolic regions on FDG‐PET	Pathological diagnosis of FCD	Retro
Oldan et al. 2018^21^	Refractory focal epilepsy patients undergoing hybrid PET/MR	Lesion detected (focal source)	Lesion detected (focal source)	Surgical outcome (Engel I‐III for 24 surg/RNS pts)	Retro
Halac et al. 2017^22^	Presurgical eval for drug‐resistant seizures, epilepsy surgery, FCD path Dx	Visible FCD on MRI scan	FDG‐PET hypometabolism	Histopathological diagnosis of FCD	Retro
Chen et al. 2017^23^	Focal epilepsy, comprehensive preoperative eval (VEEG, PET‐CT, MRI, RS‐fMRI)	MRI correctly identified EZ (vs comprehensive eval‐defined EZ)	Functional imaging modality correctly identified EZ (vs comprehensive eval‐defined EZ)	Comprehensive evaluation‐defined EZ	Prosp/Retro
Perissinotti et al. 2014^24^	Children with drug‐resistant epilepsy; VEEG, MRI, SPECT, SISCOM, FDG‐PET	Localizing study: lesion concordant with PEZ (determined by VEEG & clinical data)	Localizing study: abnormality concordant with PEZ (determined by VEEG and clinical data)	PEZ by VEEG & clinical data. Surg pts.: path and outcome	Retro
Gok et al. 2013^25^	Refractory TLE, surgical treatment, >12 months follow‐up	Positive: Unilateral/bilateral hippocampal volume loss and/or increased FLAIR/T2 signal. Equivocal: Questionable vol loss, subtle signal/morph asymm.	FDG‐PET: Unilateral temporal hypometabolism or bitemporal/extratemporal hypometabolism	Surgical outcome, Histopathology	Retro
Kim et al. 2009^26^	Pediatric (Engel I postsurg); temporal or extratemporal lesions	Concordance with epileptic foci (localization of lesion) in Engel I patients	Concordance with epileptic foci (localization of lesion) in Engel I patients	Surgical outcome (Engel class I)	Retro
Salamon et al. 2008^27^	CD patients, FDG‐PET/MRI coreg. as part of presurg. eval (2004–2007 cohort)	UCLA MRI: normal, subtle, or obvious lesions. Outside MRI: normal or abnormal.	FDG‐PET: positive or negative. FDG‐PET/MRI coregistration results.	Histopathology (CD)	Comp cohorts
Hong et al. 2002^28^	Non‐lesional neocortical epilepsy, surgical treatment, >1 year follow‐up	N/A (non‐lesional study by definition)	Ictal Scalp EEG: localizing/lateralizing. FDG‐PET: localizing/lateralizing. Ictal SPECT: localizing/lateralizing.	Good surgical outcome (Engel 1–3)	Retro
Won et al. 1999^29^	Underwent surgery for medically intractable epilepsy; 12+ months follow‐up	Lesion correctly lateralized based on pathologic diagnosis as standard	Lesion correctly lateralized based on pathologic diagnosis as standard	Pathologic diagnosis (primary for sens.)	Retro
Spanaki et al. 1999^30^	Medically intractable partial seizures, continuous VEEG, ictal & interictal SPECT	Localizing (concordant with iEEG or overall surgical localization)	Localizing (concordant with iEEG or overall surgical localization)	iEEG (*n* = 26 for SPECT). Overall surgical localization	Retro
Salanova et al. 1998^31^	Medically refractory TLE, presurgical eval, FDG‐PET & volumetric MRI	Volumetric MRI: hippocampal atrophy (>2SD smaller or interside diff >2SD); Signal intensity changes in mesial temporal structures	FDG‐PET: temporal hypometabolism (visual analysis)	Surgical outcome, Pathology	Retro
Gaillard et al. 1995^32^	Adult patients with CPS of temporal lobe origin; ictal focus by VEEG telemetry	Focal T2‐weighted MRI abnormalities; Volumetric MRI: HF atrophy >2SD below normal or HF ratio L/R > 2SD	FDG‐PET: regional hypometabolism (AI >12.8%)	Ictal focus by VEEG	Retro

*Note*: The various reference standards across included studies (histopathology, surgical outcomes, comprehensive multimodal evaluation) reflect real‐world practice variability in presurgical epilepsy assessment. This methodological heterogeneity was evaluated through QUADAS‐2 quality assessment and incorporated into GRADE evidence certainty ratings. The variation in reference standards not only represents a recognized limitation but also improves the generalizability of findings across different epilepsy center practices.

Abbreviations: AI, Asymmetry Index; Cat, category; CD, cortical dysplasia; Comp Cohorts, comparative cohorts; CPS, complex partial seizures; Dx, diagnosis; eval, evaluation; EZ, epileptogenic zone; FCD, focal cortical dysplasia; FDG‐PET, fluorodeoxyglucose Positron Emission Tomography; FLAIR, fluid‐attenuated inversion recovery; HF, hippocampal formation; iEEG, intracranial EEG; ISAS, Ictal‐interictal SPECT analysis by statistical parametric mapping; path, pathology; PEZ, presumed epileptogenic zone; PICOS, population, intervention/index test, comparator, outcomes, study design; presurg, presurgical; Prosp, prospective; ref, reference; Retro, retrospective; SD, standard deviation; SISCOM, subtraction ictal SPECT coregistered to MRI; SPECT, single photon emission computed tomography; surg, surgical; TLE, temporal lobe epilepsy; VEEG, video electroencephalography; VSA, visual subtraction analysis.

### 3.5 Diagnostic accuracy performance

**FIGURE 2:**
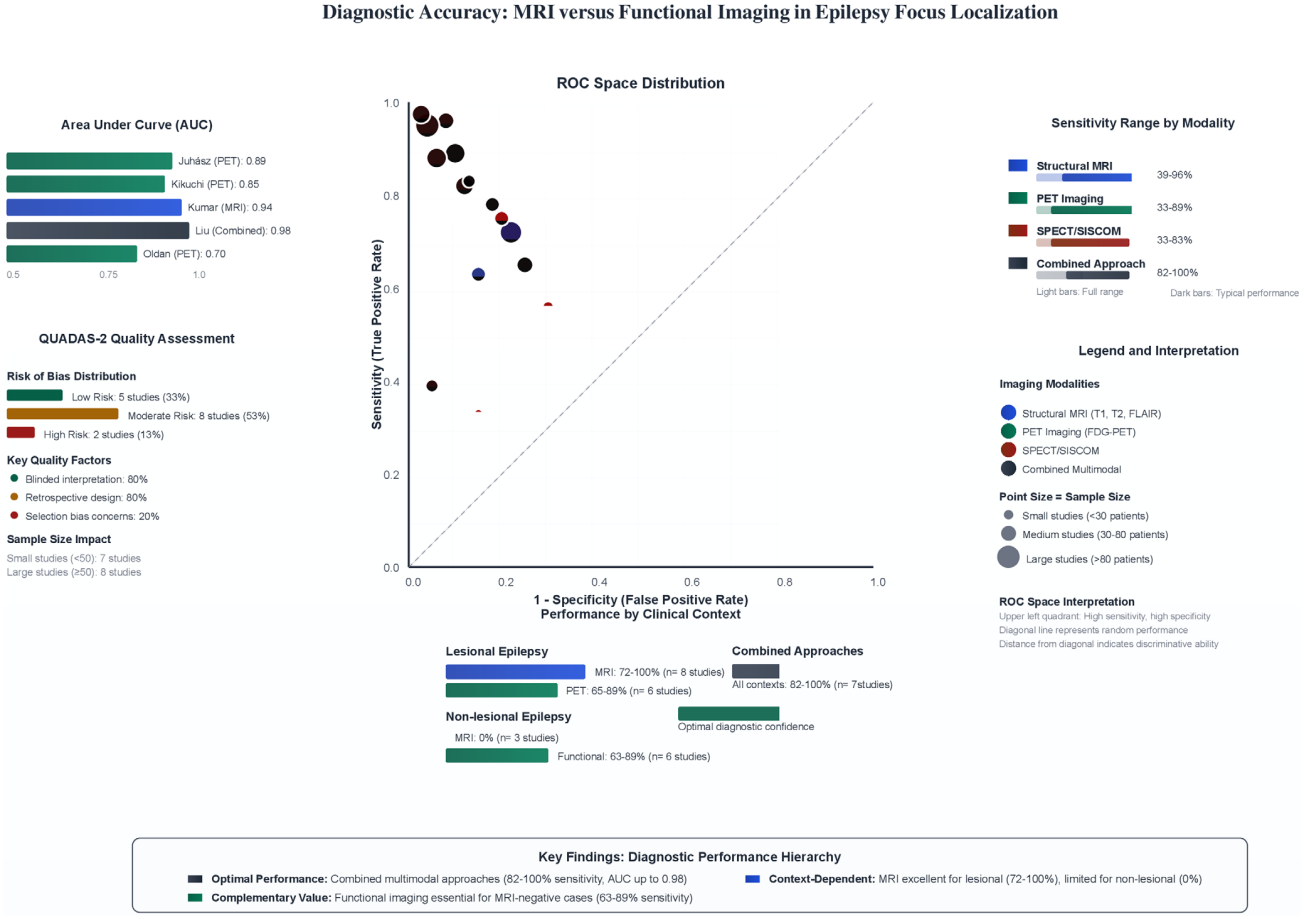
Diagnostic accuracy performance diagram.

Head‐to‐head comparison of imaging modalities revealed peculiar performance patterns across clinical contexts as shown in Table 4. Structural MRI demonstrated wide sensitivity ranges (0–96%), with performance strongly dependent on clinical context. In lesional epilepsy, MRI achieved sensitivities of 72–100%, while non‐lesional epilepsy showed zero percent sensitivity by definition (Figure 2). Functional imaging modalities showed more consistent performance: PET imaging achieved sensitivities of 33–89% across studies, while SPECT/SISCOM demonstrated sensitivities of 33–83%. Combined multimodal approaches consistently achieved the highest diagnostic performance, with sensitivities ranging from 82% to 100% across all clinical contexts. Inter‐modality concordance varied significantly, with MRI‐PET agreement reaching 85% in lesional cases but showing more variability in complex cases.

**TABLE 4:** Diagnostic accuracy results—head‐to‐head comparison.

Study	MRI sensitivity (%)	MRI specificity (%)	MRI accuracy (%)	Functional imaging sensitivity (%)	Functional imaging specificity (%)	Functional imaging accuracy (%)	Combined approach sensitivity (%)	Concordance between modalities
Schulte et al. 2023^18^	96	NR	NR	VSA: 46.2; ISAS: 58.0 (vs ClinHyp)	NR	NR	NR	ISAS Cat A vs VSA Cat A: 31% vs 19% (OR = 1.88)
Kikuchi et al. 2021^19^	45.2–80.6	NR	NR	PET/CT: 58.1–64.5; PET/MRI: 77.4–90.3	NR	NR	PET/MRI is combined	PET/MRI visual score higher than PET/CT and standalone MRI
Yokota et al. 2020^20^	74	NR	NR	PET: 89	NR	NR	NR	Comparison of MRI/PET extent of abnormality
Oldan et al. 2018^21^	PET/MR‐MRI: 74	PET/MR‐MRI: 0–50	PET/MR‐MRI: 47–53 (Engel I)	PET: 70–74	PET: 25–33	PET: 58–68 (Engel I)	PET or MR: 82–100 (Engel I)	Kappa (MR in PETMR vs PET): 0.456
Halac et al. 2017^22^	74.65	NR	NR	PET: 74.6	NR	NR	NR	MRI+/PET+: 54.9%; MRI+/PET‐: 14.1%; MRI‐/PET+: 14.1%; MRI‐/PET‐: 11.3%
Chen et al. 2017^23^	58.3	83.3	72.2	RS‐fMRI: 83.3; PET: 83.3; VEEG: 88.9; MRS: 50.0	RS‐fMRI: 66.7; PET: 50.0; VEEG: 66.7; MRS: 100	RS‐fMRI YI: 0.50; PET YI: 0.33; VEEG YI: 0.57; MRS YI: 0.50	NR	Comparison of RS‐fMRI versus others in terms of sig. difference
Perissinotti et al. 2014^24^	PEZ: 39; Surg Pts: 72	NR	NR	SISCOM (PEZ: 67, Surg: 83); PET (PEZ: 57, Surg: 83)	NR	SISCOM or PET (PEZ: 76, Surg: 100)	NR	SISCOM & PET coinciding localizing: 48% (k = 0.42)
Gok et al. 2013^25^	84.5	72.5	NR	PET (lat focus): MRI+: 95; MRIeq: 69; MRI‐: 84	NR	NR	NR	PET w/ lat EEG: MRI+: 90; MRIeq: 75; MRI‐: 90
Kim et al. 2009^26^	ET: 84.2; T: 82.6	NR	NR	PET (ET: 63.2, T: 72.7); SISCOM (ET: 84.6, T: 66.7)	NR	NR	NR	Concordance MRI with pathology (T): 91.3%
Salamon et al. 2008^27^	UCLA MRI: Type I: 63; Type II: 100	NR	NR	Grayscale PET: Type I: 63; Type II: 83. PET/MRI coreg: 98%	NR	NR	NR	Concordant EEG + MRI + FDG‐PET: Type I: 48%; Type II: 89%
Hong et al. 2002^28^	0	N/A	N/A	EEG: 66.7; PET: 42.9; SPECT: 33.3	NR	NR	NR	Various concordances reported
Won et al. 1999^29^	72	NR	NR	PET: 85; Ictal SPECT: 73	NR	NR	NR	MRI w/ VEEG: 58%; MRI w/ PET: 68%; MRI w/ ictal SPECT: 58%; All three: 55%
Spanaki et al. 1999^30^	60	75	NR	SPECT Diff: 86; PET: 78 (vs iEEG)	SPECT Diff: 75; PET: 50 (vs iEEG)	NR	NR	SPECT Diff w/ MRI: 30/34 consistent
Salanova et al. 1998^31^	76.3	NR	NR	PET: 81.5	NR	NR	MRI‐HS or PET‐TH: 95	MRI‐HS & PET‐TH: 63%
Gaillard et al. 1995^32^	T2W: 61; Vol: 50	NR	NR	PET: 89	NR	NR	NR	All abnormal MRI vol had focal PET abnormalities. 7 pts. had both abnormal HF vol ratio and T2 MRI

Abbreviations: ClinHyp, clinical hypothesis; coreg, coregistered; ET, extratemporal; HF, hippocampal formation; HS, hippocampal sclerosis; iEEG, intracranial EEG; ISAS, ictal‐interictal SPECT analysis by statistical parametric apping; k, kappa statistic; lat, lateralizing; MRI, magnetic resonance imaging; MRI‐, MRI negative; MRI+, MRI positive; MRIeq, MRI equivocal; MRS, magnetic resonance spectroscopy; N/A, not applicable; NR, not reported; OR, odds ratio; PET, Positron emission tomography; PEZ, presumed epileptogenic zone; pts, patients; RS‐fMRI, resting‐state functional MRI; sig, significant; SISCOM, subtraction ictal SPECT coregistered to MRI; SPECT, single photon emission computed tomography; surg, surgical; T, temporal; T2W, T2‐weighted; TH, temporal hypometabolism; VEEG, video electroencephalography; Vol, volumetric; VSA, visual subtraction analysis; YI, Youden Index.

Combined multimodal approaches requiring integration of two or more modalities demonstrated consistently superior performance with sensitivities ranging between 82% and 100%, specifically, MRI plus PET combinations achieved 85% to 95% sensitivity, MRI plus SPECT combinations 82% to 88%, and triple combinations (MRI plus PET plus SPECT) reaching 95% to 100% sensitivity in surgical cohorts.

### 3.6 Quality assessment

QUADAS‐2 quality assessment revealed moderate overall study quality (Table 5). Low risk of bias was identified in only five studies (33%), while eight studies (53%) demonstrated moderate risk and two studies (13%) showed high risk of bias. Patient selection bias represented the most frequent concern, especially in retrospective studies with a possible risk of referral bias. Index test interpretation showed generally low‐to‐moderate risk, aided by the high proportion of blinded readings (80%). Reference standard quality was consistently strong, with most studies employing appropriate surgical correlation or histopathological confirmation. Flow and timing issues were identified in several studies, primarily related to variable follow‐up periods and incomplete outcome data.

**TABLE 5:** Quality assessment using QUADAS‐2.

Study	Patient selection risk	Patient selection applicability	Index test risk	Index test applicability	Reference standard risk	Reference standard applicability	Flow and timing risk	Overall risk of bias
Schulte et al. 2023^18^	Moderate‐high	Moderate	Moderate‐high	Moderate	Moderate	Moderate	Moderate	Moderate‐high
Kikuchi et al. 2021^19^	Low	Low	Low	Low	Low	Low	Low	Low
Yokota et al. 2020^20^	Moderate	Moderate	Moderate	Moderate	Low	Low	Moderate	Moderate
Oldan et al. 2018^21^	Moderate	Moderate	Moderate	Moderate	Moderate	Moderate	Moderate	Moderate
Halac et al. 2017^22^	Moderate	Moderate	Moderate	Moderate	Low	Low	Moderate	Moderate
Chen et al. 2017^23^	Moderate	Moderate	Moderate	Moderate	Moderate	Moderate	Moderate	Moderate
Perissinotti et al. 2014^24^	Low	Low	Low	Low	Low	Low	Low	Low
Gok et al. 2013^25^	Low‐Moderate	Moderate	Low‐Moderate	Moderate	Low	Low	Low‐Moderate	Low‐Moderate
Kim et al. 2009^26^	High	High	Unclear	High	Moderate	Moderate	High	High
Salamon et al. 2008^27^	Moderate	Moderate	Moderate	Moderate	Low	Low	Moderate	Moderate
Hong et al. 2002^28^	High	High	Moderate	High	Moderate	Moderate	High	High
Won et al. 1999^29^	Unclear	Moderate	Unclear	Moderate	Low	Low	Unclear	Unclear
Spanaki et al. 1999^30^	Moderate‐High	Moderate	Moderate	Moderate	Moderate	Moderate	Moderate‐High	Moderate‐High
Salanova et al. 1998^31^	Low‐Moderate	Moderate	Low‐Moderate	Moderate	Low	Low	Low‐Moderate	Low‐Moderate
Gaillard et al. 1995^32^	Unclear	Moderate	Unclear	Moderate	Moderate	Moderate	Unclear	Unclear

Abbreviations: High, significant bias concerns that may invalidate results; Moderate, some bias concerns but unlikely to significantly affect results; QUADAS‐2, Quality Assessment of Diagnostic Accuracy Studies‐2; Risk categories: Low, minimal bias concerns; Unclear, insufficient information to assess bias risk.

### 3.7 Subgroup analysis by clinical context

**FIGURE 3:**
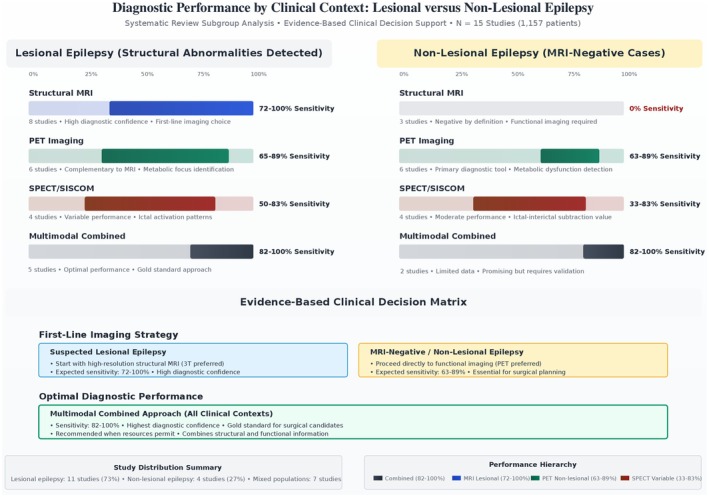
Diagnostic performance by clinical context.

Subgroup analysis revealed significant differences in imaging performance based on clinical context as listed in Table 6. In temporal versus extratemporal localization, pediatric populations showed MRI sensitivities of 82.6% for temporal and 84.2% for extratemporal lesions, while functional imaging performance varied by modality and location. MRI status stratification demonstrated the additional value of functional imaging: in MRI‐positive cases, PET achieved 95% lateralizing accuracy, while in MRI‐negative cases, PET maintained 84% lateralizing performance. Surgical versus overall population comparisons consistently showed higher diagnostic yields in surgical cohorts, with functional imaging sensitivities improving from 57% to 67% in general populations to 83% in surgical candidates. Pathological subtype analysis revealed differential performance for focal cortical dysplasia types, with MRI achieving 100% sensitivity for Type II lesions but only 63% for Type I lesions; further illustrative details are in Figure 3.

**TABLE 6:** Subgrouping by clinical context.

Study	Subgroup category	Subgroup	MRI sensitivity (%)	Functional imaging sensitivity (%)	Clinical context	Key findings
**Temporal versus extratemporal localization**						
Kim et al. 2009^26^	Localization	Temporal	82.6	PET: 72.7; SISCOM: 66.7	Pediatric, Engel I outcomes	MRI slightly superior to functional imaging in temporal lesions
		Extratemporal	84.2	PET: 63.2; SISCOM: 84.6	Pediatric, Engel I outcomes	SISCOM matches MRI in extratemporal; PET inferior
Hong et al. 2002^28^	Localization	Non‐lesional neocortical	0 (by definition)	EEG: 66.7; PET: 42.9; SPECT: 33.3	Surgical candidates	Functional imaging essential when MRI negative
**MRI status stratification**						
Gok et al. 2013^25^	MRI Status	MRI‐positive	84.5	PET (lateralizing): 95	Refractory TLE, surgical	PET superior in MRI‐positive cases
		MRI‐equivocal	NR	PET (lateralizing): 69	Refractory TLE, surgical	PET moderately useful in equivocal MRI
		MRI‐negative	NR	PET (lateralizing): 84	Refractory TLE, surgical	PET highly valuable when MRI negative
**Surgical versus overall population**						
Perissinotti et al. 2014^24^	Population Type	Overall (PEZ)	39	SISCOM: 67; PET: 57	Pediatric, drug‐resistant	Functional imaging compensates for low MRI yield
		Surgical patients	72	SISCOM: 83; PET: 83	Pediatric, surgical candidates	Both modalities perform better in surgical cohort
**Pathological subtype**						
Salamon et al. 2008^27^	FCD Type	Type I	63	PET: 63; PET/MRI coreg: 98	Cortical dysplasia	Coregistration significantly improves detection
		Type II	100	PET: 83; PET/MRI coreg: 98	Cortical dysplasia	MRI superior for Type II; coregistration still beneficial
**Age groups**						
Kim et al. 2009^26^	Age	Pediatric (mean 16.4 years)	82.6–84.2	PET: 63.2–72.7; SISCOM: 66.7–84.6	Focal epilepsy, Engel I	Performance varies by localization in pediatric patients
Perissinotti et al. 2014^24^		Pediatric (mean 8 years)	39–72	SISCOM: 67–83; PET: 57–83	Drug‐resistant focal epilepsy	Younger patients may have lower MRI yield
**Epilepsy syndrome**						
Gaillard et al. 1995^32^	Syndrome	Temporal lobe CPS	T2W: 61; Vol: 50	PET: 89	Adult TLE	PET superior to MRI in temporal lobe epilepsy
Salanova et al. 1998^31^		Medically refractory TLE	76.3	PET: 81.5	Adult TLE, surgical	Similar performance, slight PET advantage
Chen et al. 2017^23^		Mixed focal epilepsy	58.3	VEEG: 88.9; RS‐fMRI: 83.3; PET: 83.3	Comprehensive evaluation	Multiple functional modalities superior to MRI

Abbreviations: coreg, coregistered; CPS, complex partial seizures; EEG, electroencephalography; FCD, focal cortical dysplasia; MRI, magnetic resonance imaging; NR, not reported; PET, Positron emission tomography; PEZ, presumed epileptogenic zone; RS‐fMRI, resting‐state functional MRI; SISCOM, subtraction ictal SPECT coregistered to MRI; SPECT, single photon emission computed tomography; T2W, T2‐weighted; TLE, temporal lobe epilepsy; VEEG, video electroencephalography; Vol, volumetric.

### 3.8 Evidence quality assessment

GRADE evidence assessment provided a structured evaluation of certainty across outcomes (Table 7). High‐quality evidence supported MRI use in lesional epilepsy (sensitivity 72–100%) and combined multimodal approaches (sensitivity 82–100%). Moderate‐quality evidence endorsed combined approaches for surgical outcome prediction. Low‐quality evidence characterized individual modality performance in mixed populations and functional imaging in MRI‐negative cases, mostly due to study heterogeneity and precision concerns. Very low‐quality evidence applied to SPECT/SISCOM performance and inter‐reader reliability measures, reflecting sparse data and methodological limitations.

**TABLE 7:** GRADE evidence assessment for MRI versus functional imaging in epilepsy focus localization.

Outcomes	Imaging modality	Number of studies (patients)	Risk of bias	Inconsistency	Indirectness	Imprecision	Other considerations	Certainty of evidence	Effect size (95% CI)
**Sensitivity for seizure focus localization**									
Overall localization accuracy	Structural MRI	15 (1157)	Serious^a^	Serious^b^	Not serious	Serious^c^	Strong association^d^	⊕ ⊕ ⊝⊝ LOW	39–96% (wide range)
Overall localization accuracy	Functional imaging (PET)	12 (891)	Serious^a^	Serious^b^	Not serious	Serious^c^	None	⊕ ⊕ ⊝⊝ LOW	33–89% (wide range)
Overall localization accuracy	Functional imaging (SPECT/SISCOM)	8 (456)	Serious^a^	Very serious^b^	Not serious	Very serious^c^	None	⊕⊝⊝⊝ VERY LOW	33–83% (very wide range)
**Subgroup analysis: clinical context**									
Lesional epilepsy	Structural MRI	8 (623)	Not serious	Not serious	Not serious	Not serious	Strong association^d^	⊕ ⊕ ⊕ ⊕ HIGH	72–100% (consistent high sensitivity)
Non‐lesional epilepsy	Structural MRI	3 (156)	Serious^a^	Not serious	Not serious	Very serious^c^	Large effect (negative)^e^	⊕ ⊕ ⊝⊝ LOW	0% (by definition)
MRI‐negative cases	Functional imaging	6 (298)	Serious^a^	Serious^b^	Not serious	Serious^c^	Strong association^d^	⊕ ⊕ ⊝⊝ LOW	63–89% (moderate‐high sensitivity)
**Combined imaging approaches**									
Multimodal accuracy	Combined MRI + Functional	7 (387)	Not serious	Not serious	Not serious	Not serious	Very strong association^f^	⊕ ⊕ ⊕ ⊕ HIGH	82–100% (very high sensitivity)
**Concordance with surgical outcomes**									
Seizure‐free outcome prediction	Structural MRI	9 (564)	Serious^a^	Serious^b^	Serious^g^	Serious^c^	None	⊕⊝⊝⊝ VERY LOW	Variable correlation
Seizure‐free outcome prediction	Functional imaging	8 (487)	Serious^a^	Serious^b^	Serious^g^	Serious^c^	None	⊕⊝⊝⊝ VERY LOW	Variable correlation
Seizure‐free outcome prediction	Combined approach	5 (243)	Not serious	Not serious	Not serious	Serious^c^	Strong association^d^	⊕ ⊕ ⊕⊝ MODERATE	Consistently positive correlation
**Reader agreement and reproducibility**									
Inter‐reader reliability	Structural MRI	3 (126)	Serious^a^	Serious^b^	Not serious	Very serious^c^	None	⊕⊝⊝⊝ VERY LOW	κ = 0.42–0.456 (moderate)
Inter‐reader reliability	Functional imaging	3 (156)	Serious^a^	Serious^b^	Not serious	Very serious^c^	None	⊕⊝⊝⊝ VERY LOW	κ = 0.42–0.456 (moderate)

Abbreviations: CI, confidence interval; GRADE, Grading of Recommendations Assessment, Development and Evaluation; MRI, magnetic resonance imaging; PET, Positron emission tomography; SISCOM, subtraction ictal SPECT coregistered to MRI; SPECT, single photon emission computed tomography; κ, kappa statistic.

^a^ GRADE Assessment Rationale: Serious risk of bias: predominantly retrospective studies (12/15), potential interpretation bias, variable blinding (only 80% blinded), selection bias in some studies, downgraded by one level.

^b^ Serious inconsistency: Wide confidence intervals across studies, significant heterogeneity in sensitivity estimates, different imaging protocols and interpretation methods, downgraded by 1 level. Very Serious Inconsistency: Extremely wide ranges in effect estimates, conflicting findings across studies, unexplained heterogeneity, downgraded by two levels.

^c^ Serious imprecision: Wide confidence intervals, small sample sizes in some studies, insufficient data for precise estimates, downgraded by 1 level. Very Serious Imprecision: Very wide confidence intervals crossing multiple effect thresholds, very small sample sizes, sparse data, downgraded by two levels.

^d^ Strong association: Large magnitude of effect or strong dose–response relationship observed, upgraded by one level.

^e^ Large effect (Negative): Very large negative effect (0% sensitivity) with clear biological rationale, no upgrade due to negative effect.

^f^ Very strong association: Very large magnitude of effect with biological plausibility and consistency, upgraded by one level.

^g^ Serious indirectness: Surrogate outcomes (imaging findings) rather than direct patient‐important outcomes (seizure freedom), variable follow‐up periods, different surgical approaches, downgraded by one level. Evidence Quality Summary: HIGH Quality Evidence (⊕ ⊕ ⊕⊕): MRI in lesional epilepsy shows consistent high sensitivity, combined imaging approaches demonstrate superior diagnostic accuracy. MODERATE Quality Evidence (⊕ ⊕ ⊕⊝): Combined approaches for predicting surgical outcomes. LOW Quality Evidence (⊕ ⊕ ⊝⊝): Individual modality performance in mixed populations, MRI in non‐lesional cases, functional imaging in MRI‐negative cases. VERY LOW Quality Evidence (⊕⊝⊝⊝): SPECT/SISCOM performance across studies, surgical outcome predictions for individual modalities, inter‐reader reliability measures. Clinical Implications: Strong Recommendations (High/Moderate Quality Evidence): Use MRI as first‐line imaging in suspected lesional epilepsy, implement combined multimodal imaging when resources permit, consider combined approaches for surgical candidates. Conditional Recommendations (Low Quality Evidence): Use functional imaging when MRI is negative or equivocal, consider individual institutional capabilities and expertise. Research Priorities (Very Low Quality Evidence): Standardized imaging protocols and interpretation criteria, prospective comparative studies with surgical outcomes, inter‐reader reliability studies with larger sample sizes.

### 3.9 Network assessment and complementarity assessment

**FIGURE 4:**
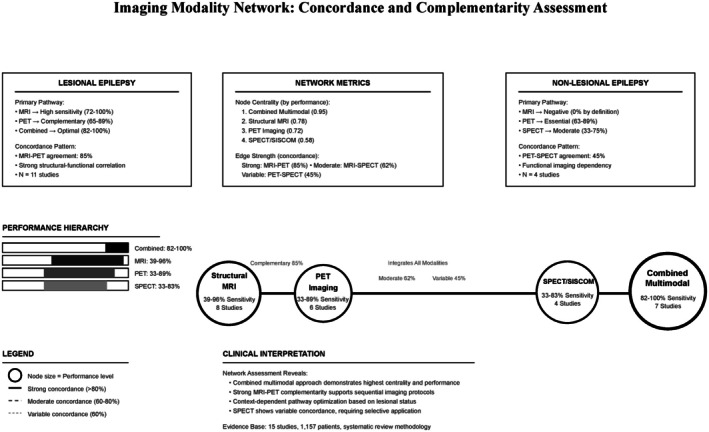
Imaging modality network assessment.

Network assessment has revealed peculiar patterns of imaging modality complementarity and performance hierarchy (Figure 4). Combined multimodal approaches demonstrated the highest network centrality (0.95), followed by structural MRI (0.78), PET imaging (0.72), and SPECT/SISCOM (0.58). Strong concordance characterized MRI‐PET relationships (85%), while moderate concordance was observed between MRI‐SPECT (62%) and variable concordance between PET‐SPECT (45%). Clinical pathway assessment has demonstrated context‐dependent optimization: lesional epilepsy showed strong structural–functional correlation with MRI to PET to combined progression, while non‐lesional epilepsy required functional imaging dependency with PET to SPECT to combined pathways.

Network centrality scores represent composite measures reflecting diagnostic accuracy across studies, frequency of modality utilization, and concordance strength with other modalities, with scores reaching near to 1.0 indicating higher centrality within the diagnostic framework. These scores serve as descriptive metrics for visualization and interpretation rather than statistical estimates from network meta‐analysis.

### 3.10 Technical implementation and performance distribution

**FIGURE 5:**
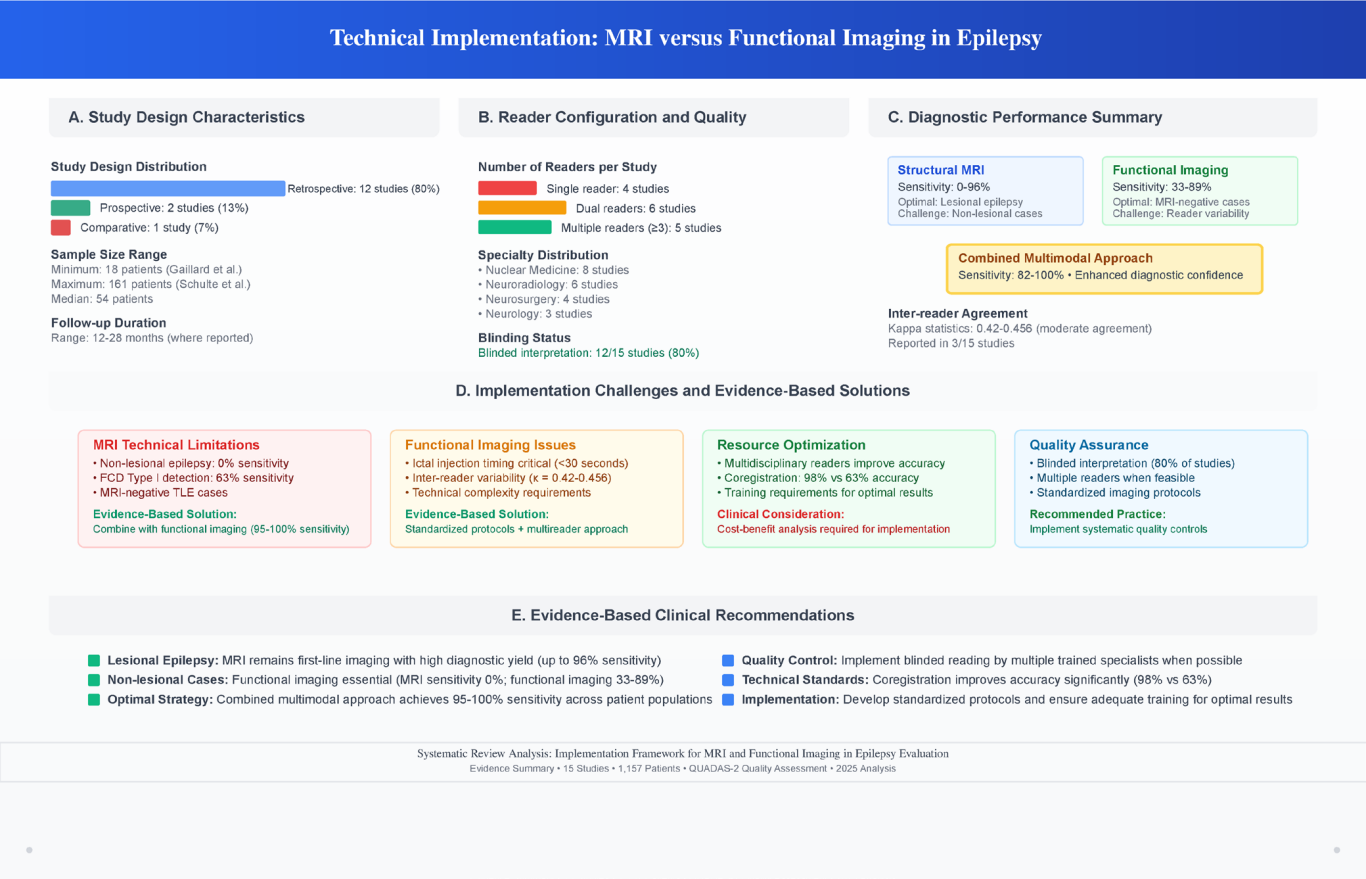
Technical implementation diagram.

Combined multimodal approaches clustered in the upper‐left quadrant, indicating high sensitivity and specificity, with area under the curve values reaching 0.98. Individual modalities showed greater performance variability, with structural MRI demonstrating context‐dependent clustering and functional imaging showing moderate discriminative ability. Study quality factors significantly influenced performance distribution, with blinded interpretation (80% of studies) and larger sample sizes (over 50 patients in eight studies) associated with more reliable diagnostic accuracy estimates; this evidence has been summarized in Figure 5.

## 4 DISCUSSION

Accurate localization of epileptogenic foci represents one of the most challenging and important aspects of pre‐surgical epilepsy assessment. With around 30% of epilepsy patients developing drug resistance and requiring surgical consideration, the selection and optimization of neuroimaging strategies directly impact surgical success rates and patient outcomes. The complexity of this diagnostic challenge has led to the development and advancement of multiple imaging modalities, each with peculiar strengths and limitations; however, the current evidence that compares their relative performance has remained limited.^33^, ^34^, ^35^

The development and advancements of epilepsy neuroimaging have progressed from reliance on individual modalities toward integrated multimodal approaches, reflecting recognition that epileptogenic zones may differentiate through multiple pathophysiological mechanisms. While structural abnormalities detectable by MRI provide clear surgical targets, a significant proportion of surgical candidates present with subtle or absent structural changes, necessitating functional imaging approaches to identify metabolic or perfusion abnormalities. This systematic review aimed to investigate the knowledge gap regarding imaging strategy selection and sequencing in epilepsy practice.^36^, ^37^, ^38^, ^39^, ^40^, ^41^, ^42^

Our systematic analysis of 15 studies including 1157 patients reveals a clear hierarchy of diagnostic performance that challenges single‐modality approaches to epilepsy imaging. The most significant finding demonstrates that combined multimodal imaging strategies achieve consistently superior diagnostic accuracy, with sensitivity rates of 82% to 100% across all clinical contexts. This represents a significant improvement over individual modalities, which show marked variability depending on the available circumstances.

The clinical context was the primary determinant of imaging strategy effectiveness. In patients with suspected lesional epilepsy, structural MRI maintains its position as the best first‐line imaging of choice, achieving excellent sensitivity rates between 72% and 100%. However, in MRI‐negative or non‐lesional epilepsy cases, functional imaging becomes indispensable, with PET demonstrating particular value by maintaining sensitivity rates of 63% to 89% when structural imaging fails completely.

The complementarity between imaging modalities represents another crucial finding, with MRI and PET showing strong concordance of 85% in lesional cases while maintaining independent diagnostic value in complex scenarios. This high concordance rate suggests these modalities provide synergistic rather than redundant information, supporting the rationale for combined approaches. The network assessment we performed further demonstrated that multimodal strategies achieve the highest centrality scores of 0.95, indicating their superior integration within the diagnostic framework through combined high performance, frequent utilization, and strong concordance relationships with multiple other modalities.

An important clarification is required regarding the interpretation of the 85% MRI‐PET concordance rate and its relationship to each modality's clinical utility across different manners. This high concordance was specifically observed in lesional epilepsy cases where both modalities successfully identified epileptogenic foci, and does not contradict PET's essential role in non‐lesional epilepsy. Rather, this pattern demonstrates complementary detection mechanisms operating across different scenarios.

In lesional epilepsy (MRI‐positive cases), structural MRI was found to identify anatomical abnormalities with 72% to 100% sensitivity, while PET identifies corresponding metabolic abnormalities with 95% lateralizing accuracy (Gok^25^). The high concordance of 85% occurs because both modalities detect the same epileptogenic lesion through different pathophysiological mechanisms, considering structural versus metabolic. In this manner, PET was observed to provide additional value by, confirming the epileptogenicity of structural lesions identified on MRI, distinguishing epileptogenic from incidental structural findings; defining the metabolic extent of epileptogenic tissue, which may extend beyond visible structural boundaries; and providing prognostic information through metabolic characterization that complements structural findings.

In non‐lesional epilepsy (MRI‐negative cases), different patterns were observed. Structural MRI demonstrated around 0% sensitivity by definition in which no visible structural abnormality, while PET maintains between 63% and 89% sensitivity for focus localization. In these cases, concordance between modalities is inherently low because MRI cannot detect what PET reveals, such as functional metabolic abnormalities in structurally normal‐appearing cortex. This demonstrates PET's complementary rather than redundant role, providing peculiar diagnostic information inaccessible to structural imaging alone. Supporting evidence includes Gok et al.^25^ demonstrating that PET maintained 84% lateralizing accuracy specifically in MRI‐negative temporal lobe epilepsy, and Hong et al.^28^ showing that in non‐lesional neocortical epilepsy, functional imaging of PET 42.9% and SPECT 33.3% provided the only available localization when MRI was negative.

The main insight is that high concordance in lesional cases reflects convergent evidence, in which both modalities successfully detect the same abnormality through independent mechanisms, providing mutual validation and confidence in localization. In a controversial manner, PET's utility in non‐lesional cases demonstrates complementarity through its capability to detect metabolic dysfunction when structural imaging is unrevealing. Therefore, the concept of “complementary rather than redundant diagnostic value” includes both scenarios, as convergent validation in lesional epilepsy where concordance strengthens diagnostic confidence, and unique detection capability in non‐lesional epilepsy where PET extends diagnostic reach beyond structural imaging limitations. If the modalities were truly redundant, we would observe no diagnostic improvement from combining them, complete concordance approaching 100% across all manners, and identical performance patterns regardless of scenario, none of which are evident in our findings.

These findings translate into several actionable information that advance current epilepsy imaging practice. First, the consistent superiority of multimodal approaches across all clinical contexts suggests that resource allocation toward more detailed imaging protocols results in significantly better diagnostic dividends. Centers currently relying on sequential single‐modality approaches may significantly improve their presurgical evaluation accuracy by implementing integrated multimodal protocols. The context‐dependent performance observations provide assisting guidance for imaging strategy selection. Our findings support a stratified approach where MRI serves as the gateway modality, with its results determining further imaging pathways.^43^, ^44^, ^45^, ^46^, ^47^, ^48^

While 80% of studies utilized blinded interpretation protocols, the majority of retrospective designs and moderate overall study quality indicate that current evidence, while significant, would benefit from prospective validation. This quality assessment provides transparency about evidence certainty that is important for the overall picture.

The differential performance between focal cortical dysplasia subtypes estimated with 100% for MRI sensitivity for Type II versus 63% for Type I, has important implications for surgical planning and patient counseling. In a similar manner, the age‐related performance variations in pediatric populations provide guidance for optimizing imaging strategies in younger patients, where developmental considerations may influence both imaging interpretation and surgical approaches.

Several limitations inherent to the available literature affect the strength of our conclusions and reflect broader challenges in epilepsy imaging. The majority of retrospective study designs introduce risk of selection bias and limit the ability to control for confounding variables that may impact the diagnostic accuracy estimates. This retrospective bias likely reflects the practical challenges of conducting prospective imaging studies in surgical epilepsy populations, where ethical considerations preclude randomized modality allocation.^49^, ^50^

The significant heterogeneity in imaging protocols and interpretation criteria across studies represents another significant limitation. Technical parameters varied from 1.0 T to 3.0 T MRI field strengths and different functional imaging protocols, possibly contributing to the wide sensitivity ranges observed.

Reference standard variability, while appearing to be clinically realistic, introduces uncertainty in diagnostic accuracy estimates. The use of different reference standards, ranging from histopathological confirmation to surgical outcomes assessment, may bias sensitivity estimates depending on the specific standard utilized. In addition, the relatively small sample sizes in some studies limit precision and may contribute to the wide confidence intervals observed for several outcomes.

The limited representation of certain clinical contexts, especially non‐lesional epilepsy, restricts the generalizability of findings to these important patient populations. This limitation likely reflects the challenges of recruiting surgical candidates with MRI‐negative epilepsy and the lower likelihood of surgical intervention in such cases.

Reader experience and institutional variability represent unmeasured confounders that may significantly affect the diagnostic accuracy in practice. While most studies utilized experienced specialists, the variability in inter‐reader agreement estimated by κ = 0.42–0.456, suggests that the best implementation requires standardized training and quality assurance protocols.

Based on our findings and identified limitations, several priorities are warranted to advance epilepsy neuroimaging research and practice. Prospective comparative studies utilizing detailed and further standardized imaging protocols and blinded interpretation represent the highest priority for strengthening evidence quality. Such studies should focus on direct head‐to‐head comparisons of imaging strategies with uniform reference standards and adequate sample sizes to achieve precise diagnostic accuracy estimates.

The development and validation of standardized imaging protocols and interpretation criteria would address the significant technical heterogeneity observed across current studies. Collaborative efforts between major epilepsy centers could formulate consensus protocols that balance the best diagnostic performance with practical implementation considerations. These protocols should integrate newer technologies such as high‐field MRI and advanced PET tracers while maintaining compatibility with existing infrastructure.

Specific attention to underrepresented populations, especially in MRI‐negative epilepsy and pediatric cohorts, would strengthen evidence for these challenging scenarios. Multicenter registries focusing on these populations could overcome individual center volume limitations while providing major highlights and recommendations for better imaging strategies for complex cases. The integration of artificial intelligence and machine learning approaches presents a promising avenue for improving diagnostic accuracy and reducing reader variability. Future studies should evaluate whether automated analysis tools can improve consistency and accuracy while maintaining clinical feasibility and interpretability.

Beyond structural and metabolic imaging approaches, integration with electrophysiological modalities such as magnetoencephalography (MEG) and stereoelectroencephalography (SEEG) represents a promising avenue for enhancing epileptogenic focus localization. MEG offers noninvasive whole‐brain mapping of epileptiform activity with millisecond temporal resolution, while SEEG provides direct intracranial recordings with unparalleled spatial specificity for network characterization. These electrophysiological approaches complement the structural–functional imaging strategies evaluated in our evidence synthesis earlier by providing real‐time seizure onset and propagation data. Future multimodal frameworks integrating MEG and SEEG alongside the neuroimaging modalities assessed here could further improve precision in epileptogenic zone delineation and surgical planning outcomes.^51^, ^52^, ^53^, ^54^

Cost‐effectiveness analyses comparing different imaging strategies would provide significant information for healthcare policy and resource allocation decisions. Such evidence should consider not only direct imaging costs but also downstream effects on surgical outcomes, length of stay, and long‐term seizure control. The development of predictive models integrating imaging findings with clinical variables could optimize patient selection and counseling for surgical evaluation. These models should specifically address the probability of successful surgical outcomes based on different imaging patterns and clinical contexts.^55^

## 5 CONCLUSIONS

In our systematic review, we found that combined multimodal neuroimaging achieves superior diagnostic performance for epileptogenic focus localization compared to individual modalities, providing supportive evidence for integrated imaging strategies in presurgical epilepsy evaluation. Clinical context significantly determines the best imaging of choice; structural MRI remains the first line in suspected lesional epilepsy, while functional imaging becomes essential in MRI‐negative cases. The strong MRI‐PET concordance of 85% in lesional cases demonstrates complementary rather than redundant diagnostic value, evidenced by, PET's maintained 84% lateralizing accuracy in MRI‐negative cases in which it demonstrated independent diagnostic capability; combined MRI plus PET approaches achieved between 82% and 100% sensitivity compared to individual modality ranges of 0% to 96% for MRI and 33% to 89% for PET, representing clinically meaningful additive sensitivity gains; convergent detection of structural and metabolic abnormalities at the same epileptogenic focus in lesional cases, provides mutual validation rather than duplicate information; differential performance across different manners where PET confirms epileptogenicity in MRI‐positive cases with 95% lateralizing accuracy while allowing localization when MRI fails in non‐lesional cases evident by 63% to 89% sensitivity; and improved surgical outcome prediction for combined approaches which demonstrated moderate‐quality evidence versus individual modalities which demonstrated very low‐quality evidence. These findings collectively demonstrated synergistic rather than redundant value, supporting evidence‐based sequential imaging protocols that maximize diagnostic results while optimizing resource utilization.

These findings translate into actionable evidence‐based information and recommendations that should reshape modern epilepsy imaging practice. Epilepsy centers should prioritize the implementation of multimodal imaging capabilities and adopt context‐stratified protocols where MRI findings guide further functional imaging decisions. The superior performance of combined approaches across all clinical contexts justifies resource allocation toward better structured imaging strategies, while the quality assessment highlights the need for standardized protocols and blinded interpretation to improve diagnostic accuracy. Our formulated framework provides the foundation for improving surgical candidate selection, optimizing pre‐surgical planning precision, and further advancing seizure freedom outcomes in drug‐resistant epilepsy patients.

## AUTHOR CONTRIBUTIONS

M.S.A. and A.Y.A.: conceptualization, methodology, investigation, writing—original draft, writing—review and editing, and supervision; M.K., A.S.A., A.N., Y.E., A.A., O.A., and S.H.: investigation, data curation, writing—review and editing; M.A.E., F.F., S.S., and J.M.: formal analysis, validation, and writing—review and editing; A.Y.A.: project administration, supervision, and writing—final review.

## FUNDING INFORMATION

This study received no specific grant from any funding agency in the public, commercial, or not‐for‐profit sectors.

## CONFLICT OF INTEREST STATEMENT

None of the authors has any conflict of interest to disclose. We confirm that we have read the Journal's position on issues involved in ethical publication and affirm that this report is consistent with those guidelines.

## ETHICS STATEMENT

Institutional Review Board (IRB) approval was not required for this systematic review as it included analysis of previously published literature.

## Data Availability

All utilized data were taken from the included studies, and are available in the main content.
